# Sex-specific differences in the occurrence of *Fusobacterium nucleatum* subspecies and *Fusobacterium periodonticum* in the oral cavity

**DOI:** 10.18632/oncotarget.25042

**Published:** 2018-04-17

**Authors:** Karsten Henne, Hildegard Schilling, Mark Stoneking, Georg Conrads, Hans-Peter Horz

**Affiliations:** ^1^ Division of Oral Microbiology and Immunology, Department for Operative Dentistry, Periodontology and Preventive Dentistry, RWTH Aachen University Hospital, Aachen, Germany; ^2^ Department of Evolutionary Genetics, Max Planck Institute for Evolutionary Anthropology, Leipzig, Germany; ^3^ Institute of Medical Microbiology, RWTH Aachen University Hospital, Aachen, Germany

**Keywords:** Fusobacterium nucleatum, subspecies, colorerectal cancer, human saliva, periodontal pathogen

## Abstract

The periodontitis-associated species *Fusobacterium nucleatum* (FN) has been implicated in several extra-oral diseases, including preterm birth and colorectal cancer. Due to its genetic and phenotypic heterogeneity, FN is classified in four subspecies which may differ in their disease potential. Here we compared the prevalence of FN subspecies and the close relative *F. periodonticum* (FP) via 16S rRNA gene analysis in saliva from 100 healthy individuals (60 females, and 40 males) from eleven countries spanning five continents. By focusing on the most abundant sequence types (i.e. analysis of approximately ten clone sequences each) the average number of FN/FP subspecies per individual differed significantly between females and males, i.e. 2.93 versus 2.5, respectively (*P =* 0.043). FN subsp. fusiforme/vincentii was significantly more prevalent in females vs males, with 2.85 vs. 1.68 sequence reads per individual, respectively (*P =* 0.012). A significant age-related difference was observed in females but not in males, i.e. 2.6 subspecies on average in females ≤ 30 years vs. 3.2 in females > 30 (*P =* 0.0076). Given the link between FN and systemic disorders our findings highlight the need for microbial studies at the subspecies level to further characterize the role of periodontal pathogens in diseases that affect females and males differently, e.g. colorectal cancer.

## INTRODUCTION

*Fusobacterium nucleatum* (FN) is a resident member of the human oral cavity and as a member of the so-called orange complex plays an important role in the development and progression of periodontal disease [1]. Among the high number of periodontal pathogens, FN is the most frequent oral species found at sites of infections other than the oral cavity [2], including pneumonia [3], pyogenic liver abscess [4], sepsis [5], infective endocarditis [6], brain abscesses [7], and appendicitis [8]. FN of oral origin has also been implicated with pre-term birth [9–11]. Furthermore, there is growing evidence indicating that FN is involved in oral carcinogenesis [12] and colorectal cancer (CRC) [13–15]. Its role as a key constituent in the latter disease is corroborated by the fact, that FN is consistently associated with distant metastases from primary human CRC, including liver metastases and treatment with FN-effective antimicrobials reduces not only the FN load but also cancer cell proliferation and tumor growth [16].

Importantly, virulence properties and the potential for systemic dissemination can vary strongly among subtypes of oral species [7]. Therefore, elucidation of the disease potential of a bacterial species requires analysis at the subspecies level. Based on phenotypic and genotypic differences, FN has traditionally been divided into five subspecies, namely *F. nucleatum* subsp. *nucleatum* (FNn), *F. nucleatum* subsp. *polymorphum* (FNp), *F. nucleatum* subsp. *fusiforme* (FNf), *F. nucleatum* subsp. *vincentii* (FNv), and *F. nucleatum* subsp. *animalis* (FNa) [17–19]. Recently it has been proposed to classify FNf and FNv into one subspecies given their consistency based on the nucleotide sequences of the 16S rRNA gene, the RNA polymerase beta-subunit gene and zinc protease gene [20], as well as their spectral profiles generated by MALDI-TOF MS [21]. We here therefore address those two subspecies collectively as FNf/v.

In a previous study we examined the global distribution and heterogeneity of FN in saliva of human individuals across three continents [22]. The motivation for this study was to test the hypothesis that bacterial residents in the oral cavity can be used for distinction of human populations given the long-term co-evolution of humans and their microbes. While this hypothesis could be confirmed for another oral bacterial species co-analyzed in this study, namely *Streptococcus oralis*, the distribution pattern of FN subspecies did not reveal a geographic signature [22]. Analysis had been performed by cultivation-independent sequencing of the 16S–23S rRNA internal transcribed spacer (ITS) region with a PCR assay that targeted all FN subspecies along with the close relative *Fusobacterium periodonticum* (FP) [22]. In the current study we have extended the data set by including sequence data from saliva samples from additional geographic regions and investigated the distribution pattern in terms of sex and age. We found marked differences in the occurrence of FN subspecies and FP between female and male samples, which points at sex differences in the principal colonization pattern of these pathogens in the oral cavity, and hints at potential implications for the causation of sex-dependent diseases.

## RESULTS

**Figure 1:**
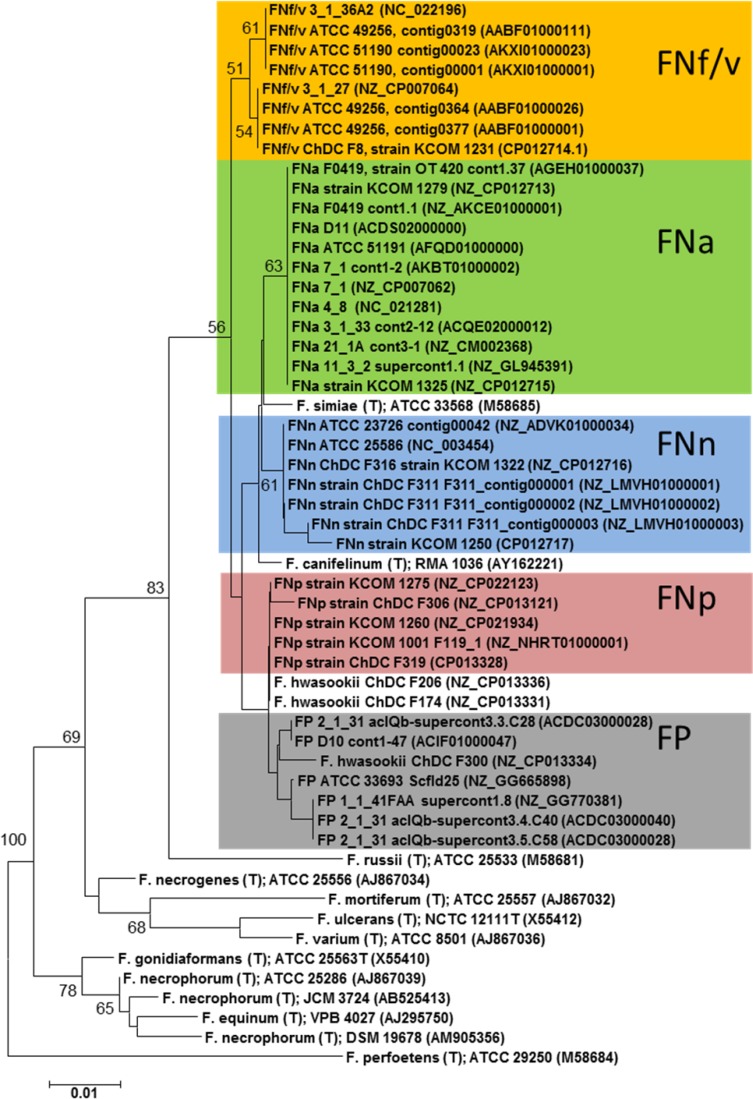
Phylogenetic relationships among subspecies of *F. nucleatum* (FN) and *F. periodonticum* (FP) in comparison with other members of the genus *Fusobacterium* The dendrogram was reconstructed based on a partial stretch of the 16S rRNA gene. Coloured sequences were extracted from publically available whole genome sequence data of well-charactized FN and FP strains. All other sequences were obtained from publically available 16S rRNA gene sequences. Sequence alignment was performed with the program MAFFT [24]. The numbers at the nodes indicate the percentage of recovery in 500 bootstrap resamplings. Only bootstrap *value*s ≥ 50% are shown. Nucleotide sequence accession-numbers are given in brackets. FNf/v: *F. nucleatum* subsp. *fusiforme/vincentii*; FNp: *F. nucleatum* subsp. *polymorphum*; FNa: *F. nucleatum* subsp. *animalis*; FNn: *F. nucleatum* subsp. *nucleatum*. The scale bar corresponds to 0.01 substitutions per nucleotide.

Using publically available reference genomes of FN subspecies and FP enabled the assignment to subspecies level based on the sequence information of a 303-bp fragment of the 16S rRNA gene (Figure 1). With aid of these reference sequences it was possible to classify the total of 1,072 16S rRNA gene sequences obtained from saliva of 100 individuals into the different FN subspecies or FP (Supplementary Figure 2). The initial collection of saliva samples was obtained from a previous study [23]. For ease of reading the different FN subspecies along with FP will henceforth be collectively addressed as “subtypes”. Highest prevalence was found for FP (91% of samples; 587 sequences), FNf/v (64% of samples; 238 sequences), FNp (55% of samples; 101 sequences), FNa (37% of samples; 59 sequences), and FNn (28% of samples, 52 sequences). No clear assignment to those recognized subtypes was possible for 35 sequences, which were not considered for further analysis in this study (Supplementary Figure 2).

**Figure 2:**
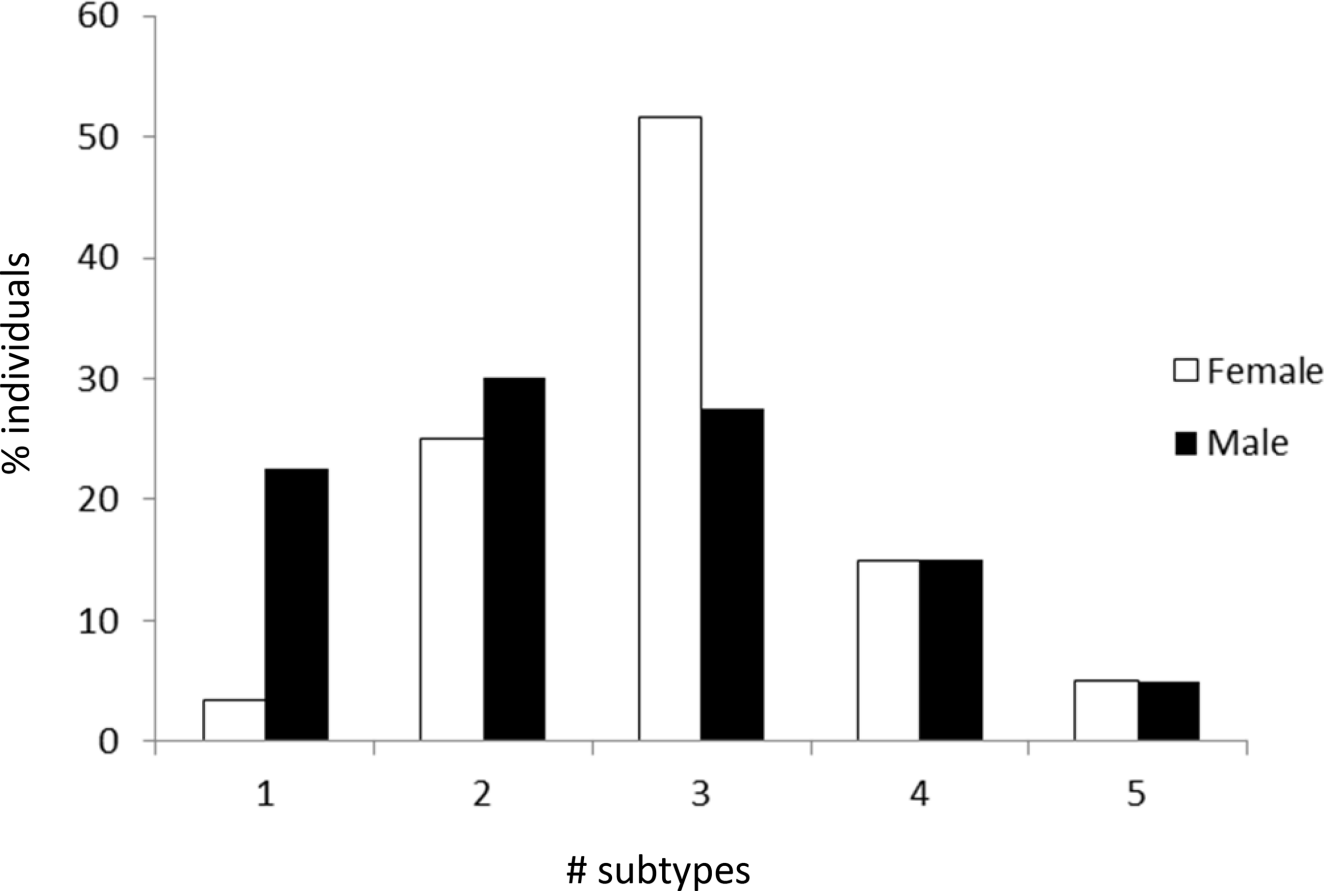
Frequency with which only one or multiple subtypes (i.e. *F. nucleatum* subspecies and *F. periodonticum*) were found in female and male individuals

**Figure 3:**
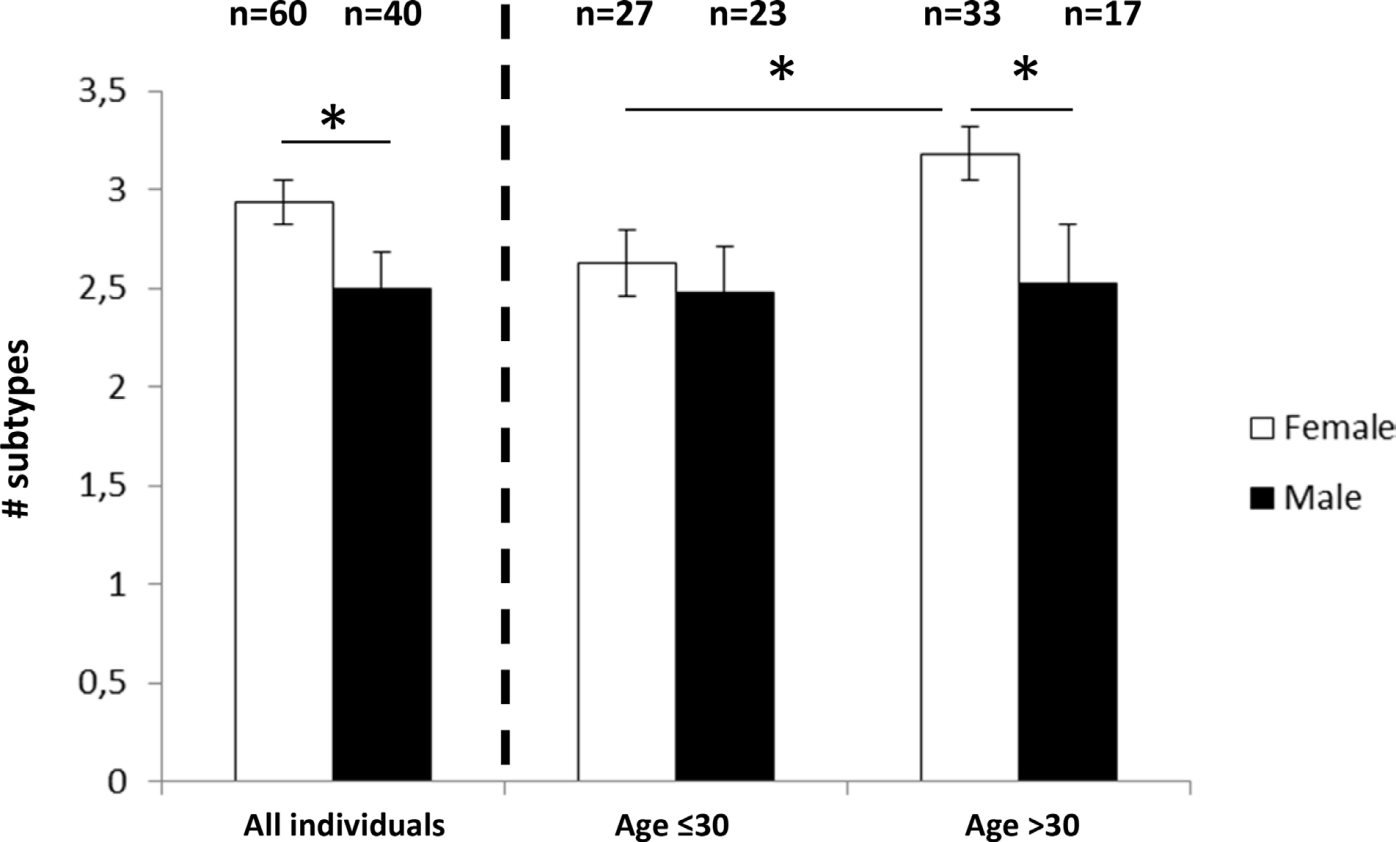
Mean number of *F. nucleatum*/*F*. *periodonticum* subtypes in female and male individuals Left: all individuals, right: grouped by age; Error bars indicate standard error. ^*^P < 0.05.

The number of subtypes detected in females differed markedly from the number of subtypes found in males (Figure 2). The average number of subtypes per individual was significantly higher in females than in males, i.e. 2.93 versus 2.5, *P =* 0.043 (Figure 3, left bars). When dividing individuals into two age categories (less than or equal to 30, and older than 30) the mean number of subtypes for the older female group was 3.2 per individual which was significantly higher compared to younger females and both age groups of males, *P <* 0.0183 (Figure 3, right bars). Hence there is an increase of subtypes with age for females, but not for males.

**Figure 4:**
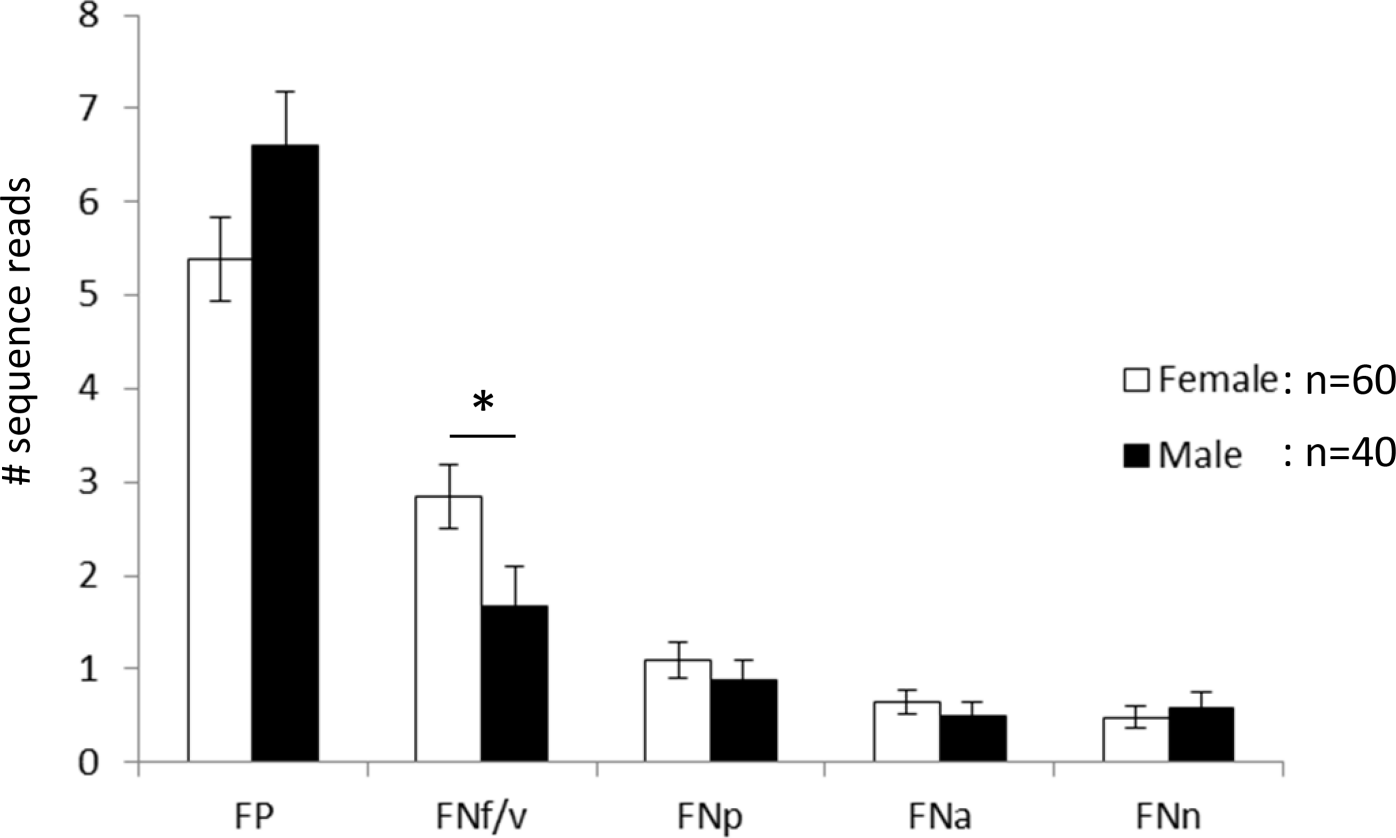
Mean number of sequence reads per individual FP: *F. periodonticum*; FNf/v: *F. nucleatum* subsp. *fusiforme*/*vincentii*; FNp: *F. nucleatum* subsp. *polymorphum*; FNa: *F. nucleatum* subsp. *animalis*; FNn: *F. nucleatum* subsp. *nucleatum*. Error bars indicate standard error. ^*^P < 0.05.

When looking at individual subtypes, a significant sex-specific difference was observed for FNf/v, which was significantly more prevalent in females than in males (i.e. 73% vs. 50%, respectively, Odds ratio: 2.75, *P =* 0.021). The average number of sequences belonging to FNf/v was 2.85 in females and 1.675 in males (*P =* 0.0117, Figure 4). There was also a trend of higher levels of FP in males versus females (*P =* 0.096, Figure 4). Otherwise no significant frequency differences between females and males were observed for the other subtypes. In addition, no age specific difference was observed for any subtype when comparing females to males.

## DISCUSSION

In the current study we performed a comparative analysis of the global incidence of FN/FP in saliva of female and male individuals. We found significant sex-specific differences, although our experimental approach was relatively insensitive, for two reasons. First, analysis of around 11 clones per individual captures only the predominant subtypes and would not detect more subtle differences between females and males. Second, the selection of individuals for sampling saliva was performed without controlling for environmental and cultural factors, such as diet, smoking or oral hygiene [22, 23]. These factors could potentially mask variation associated with sex. This means that the differential FN/FP patterns described here can be considered as robust as they are discernible with relatively shallow sequencing despite potential individual-specific variations.

Overall, we found a higher mean number of subtypes in females and an increase in the number of subtypes with age in females but not in males. Interestingly, at the subspecies level a significant difference between females and males was seen for FNf/v but this difference did not increase with age. Hence FNf/v is generally more abundant in females and not affected by attributes generally associated with aging [25]. The age-dependent increase in subtypes is thus associated with a random increase of subtypes in women. It should be noted though, that the selection of the age of 30 as a threshold was done because it split the two age groups into roughly equal numbers of individuals for both females and males. Since a correlation analysis showed only a trend for females with marginal significance (*n =* 60, *r* = 0.2251, *p =* 0.084), our data set might still be too small for drawing definite conclusions. Therefore we consider the differences of FN/FP subtypes between “younger” and “older” females as tentative which need to be further explored in a larger sample. There was also an indication of a higher prevalence and abundance of FP in males than in females; although this difference is not significant, the lack of significance could reflect the sensitivity issues discussed above. This could be addressed in a follow-up study by analyzing saliva samples adjusted for intensity of oral care, diet, smoking, ethnicity etc.

Although it is known that sex steroid hormones interact with the host immune system and with periodontal pathogens [26–28], to the best of our knowledge this is the first study that reports differences in the incidence of FN subspecies and FP between females and males. What are the implications of these observations? To begin with, there is increasing evidence that FN subspecies exert different virulence factors and are associated with different disease potential within and outside the oral cavity. FNn is thought to predominate at periodontal sites, while FNp and FNf/v are rather colonizers of healthy oral sites [29]. However this may constitute a simplified picture as subspecies-dependent interactions with other oral pathogens [30] and with the host immune-system [31] need also to be considered for assessing pathogenicity in oral infectious diseases. FP was initially isolated from severe periodontitis lesions in a juvenile diabetes patient [32], but is now generally considered a commensal with opportunistic disease potential [33]. Interestingly, recent studies even suggest a protective role of this species against caries and its potential as a predictive marker for development of this disease [34, 35]. Conversely, FP and FNp have primarily been found in samples with necrotizing ulcerative gingivitis in Chinese patients [36], indicating that no generalization can be made regarding disease potential of either subspecies.

With respect to extra-oral diseases FNa and FNp have been linked with pregnancy complications [7, 11]. In addition, FN has been implicated with colorectal cancer (CRC) [13, 37, 38]. FNa has been most frequently found in CRC specimens and this subspecies is considered a potential novel target for CRC prevention and treatment [39].

Overall the current knowledge of the precise role and importance of individual FN/FP subtypes in various diseases is still incomplete and demands further investigation. While our study does not shed additional light on the disease potential of either subtype, it points at the concept of a sex-specific consideration of bacteria involved in various human disorders. Principally, there is a recognized association between periodontal disease and a number of systemic disorders impacting females and males differently, including cancer, diabetes mellitus, rheumatoid arthritis, cardiovascular diseases, e.g. reviewed in [25]. Particularly, the list of studies supporting an association between periodontitis and cancer is large, [40–47] and excluding gynecological and breast cancer, the incidence rates for most cancers are higher in males, which is consistent across geographical regions. Notably, the incidence of CRC (in which FN is implicated) varies by sex [48–50].

In conclusion, the data presented here lends the emerging field of “gender medicine” a microbial perspective. Future studies are warranted which aim at the analysis of sex-specific microbiome analysis with resolving power at the subspecies level.

## MATERIALS AND METHODS

### Sample description

DNA-extracts from saliva samples were available from a previous study, in which sample details are described [23]. PCR amplification, cloning and sequencing of the 16S-23S rRNA internal transcribed spacer region (ITS) targeting all *F. nucleatum* subspecies and the closely related *F. periodonticum* has been described in detail in another study [22]. Those ITS sequences were retrieved from saliva samples from ten individuals each from seven geographic regions, namely China (CH), Congo (CO), Georgia (GE), Germany (DE), Philippines (PH), Poland (PO), and Turkey (TU). The purpose of that earlier study was to discern possible relationships between ITS-types and geographic locations. For a more global investigation of a sex-specific distribution, we increased the data set in the current study by sequencing the ITS from further saliva samples (again ten individuals each) from five additional countries, namely Argentina (AR), Bolivia (BO), California (CA), Louisiana (LO), and South Africa (SO). DNA extracts of those samples were also available from a previous study [23]. In the current study the ITS sequences were jointly analyzed with the following exceptions: individuals were excluded for which age was not reported (that is all ten individuals from PH and additional three individuals), and who were younger than 18 years old (four individuals). Along with three individuals for which no PCR product could be generated, this led to a total of 100 individuals (60 females and 40 males) used for analysis. The mean age was 33.5 years for females and 31.6 for males, which were not statistically different.

### Sequence analysis

On average around 11 clones were analyzed per individual to identify the most abundant subspecies. The mean number of sequence reads per individual was 10.83 for females and 10.58 for males, which were not statistically different. Table 1 and Supplementary Figure 1 summarize the sequence read statistics for all groups. Raw sequences were quality checked manually using the sequence editing software Vector NTI Advance 9.0. Sequence alignment was performed with the multiple sequence alignment program MAFFT (Multiple Alignment using Fast Fourier Transform) [24]. The assignment of sequences to FN subspecies or FP was based exclusively on the co-amplified 16S rRNA gene stretch adjacent to the ITS-region with approximately 303 bp in length, spanning the V7 and V9 regions. In order to verify the resolution power of this gene fragment, phylogenetic tree reconstruction was performed with fifty-four 16S rRNA gene fragments extracted from whole genome sequences of well characterized FN and FP strains deposited in the database of the National Center for Biotechnology Information (NCBI), https://www.ncbi.nlm.nih.gov/. Phylogenetic tree reconstruction with the neighbor-joining algorithm was performed with the computer software MEGA 6.06 (Molecular Evolutionary Genetics Analysis, release version 6140226). In order to test the reliability of the topology of the inferred phylogenetic tree the bootstrap resampling technique (500 iterations) as implemented in MEGA was used. Verification of the phylogenetic tree was also done by calculating a maximum likelihood tree and bootstrapping which confirmed the topology of the neighbor-joining tree (data not shown). Sequences used in this study are available under accession no. MH026117 - MH027188.

**Table 1:** Summary statistics of sequence reads per individual^1^

	F	M	F ≤ 30	M ≤ 30	F > 30	M > 30
***n***	60	40	27	23	33	17
**minimum # of reads per individual**	8	6	9	7	8	6
**maximum # of reads per individual**	14	15	14	15	14	13
**mean # of reads per individual**	10.83	10.58	10.96	10.74	10.73	10.35
**standard deviation**	1.24	1.60	1.19	1.66	1.28	1.54
**median**	11	11	11	11	11	11

^1^Samples are grouped in females/males and in age ≤ 30 / > 30 years; F = females, M = males.

Significant differences in the number of FN/FP subtypes between sex and age groups were assessed by the Wilcoxon-Mann-Whitney-Test. In order to test whether FNf/v was significantly more prevalent in females than in males, the Fishers exact test was used. The Spearman rank-order correlation coefficient was calculated in order to determine whether age was positively correlated with the number of FN/FP subtypes.

## SUPPLEMENTARY MATERIALS FIGURES


