# Supplementary material for: Sex-specific differences in the occurrence of Fusobacterium nucleatum subspecies and Fusobacterium periodonticum in the oral cavity
Source: Oncotarget. 2018 Apr 17;9(29):20631–9. doi: 10.18632/oncotarget.25042 (PMC5945502; doi:10.18632/oncotarget.25042)
Supplement: Supplementary file 1 [file oncotarget-09-20631-s001.pdf]

Sex-specific differences in the occurrence of *Fusobacterium nucleatum* subspecies and *Fusobacterium periodonticum* in the oral cavity

SUPPLEMENTARY MATERIALS

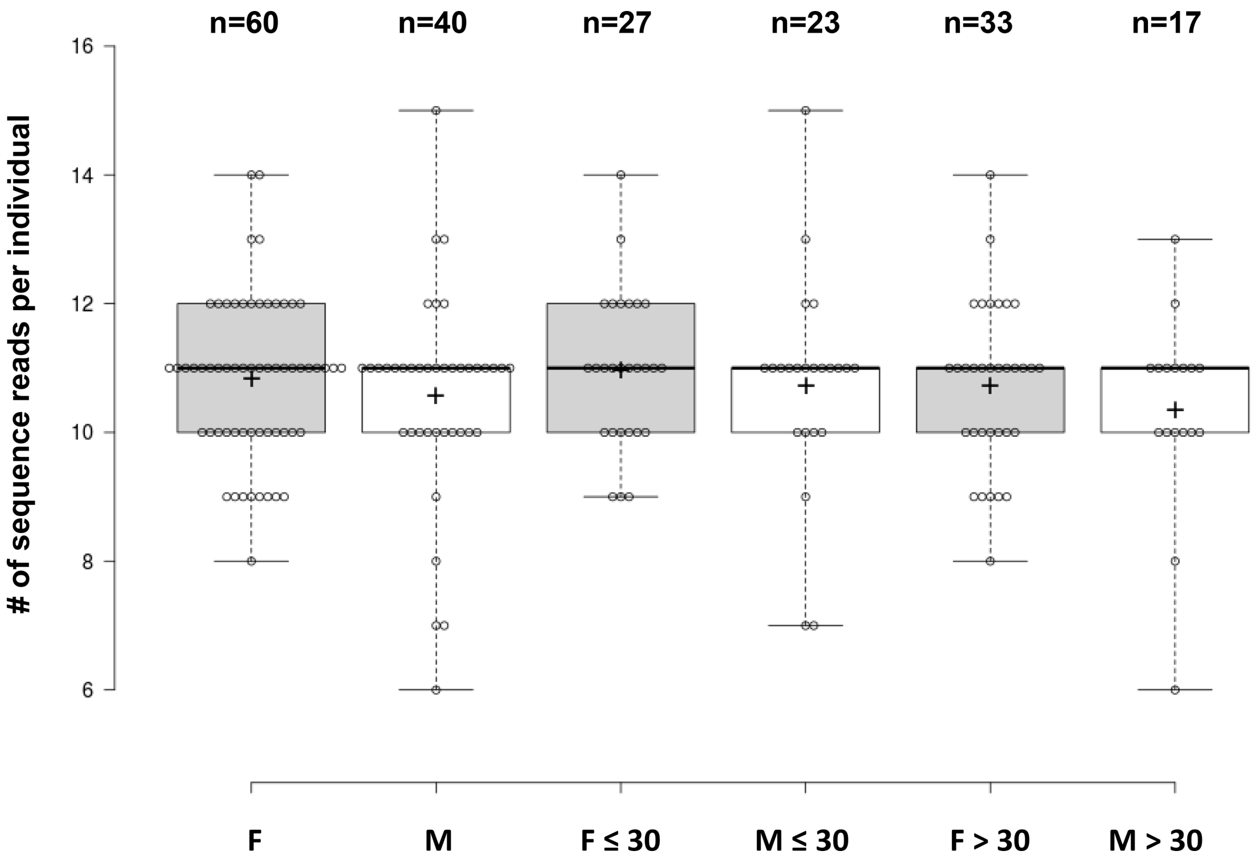

Supplementary Figure 1: Bee swarm boxplot displaying the number of sequence reads per individual. Samples are grouped in females/males and in age ≤ 30 /> 30 years. Center lines show the medians; "+" indicates the sample means; box limits indicate the 25th and 75th percentiles; whiskers extend to minimum and maximum values; data points are plotted as open circles. F = females, M = males.

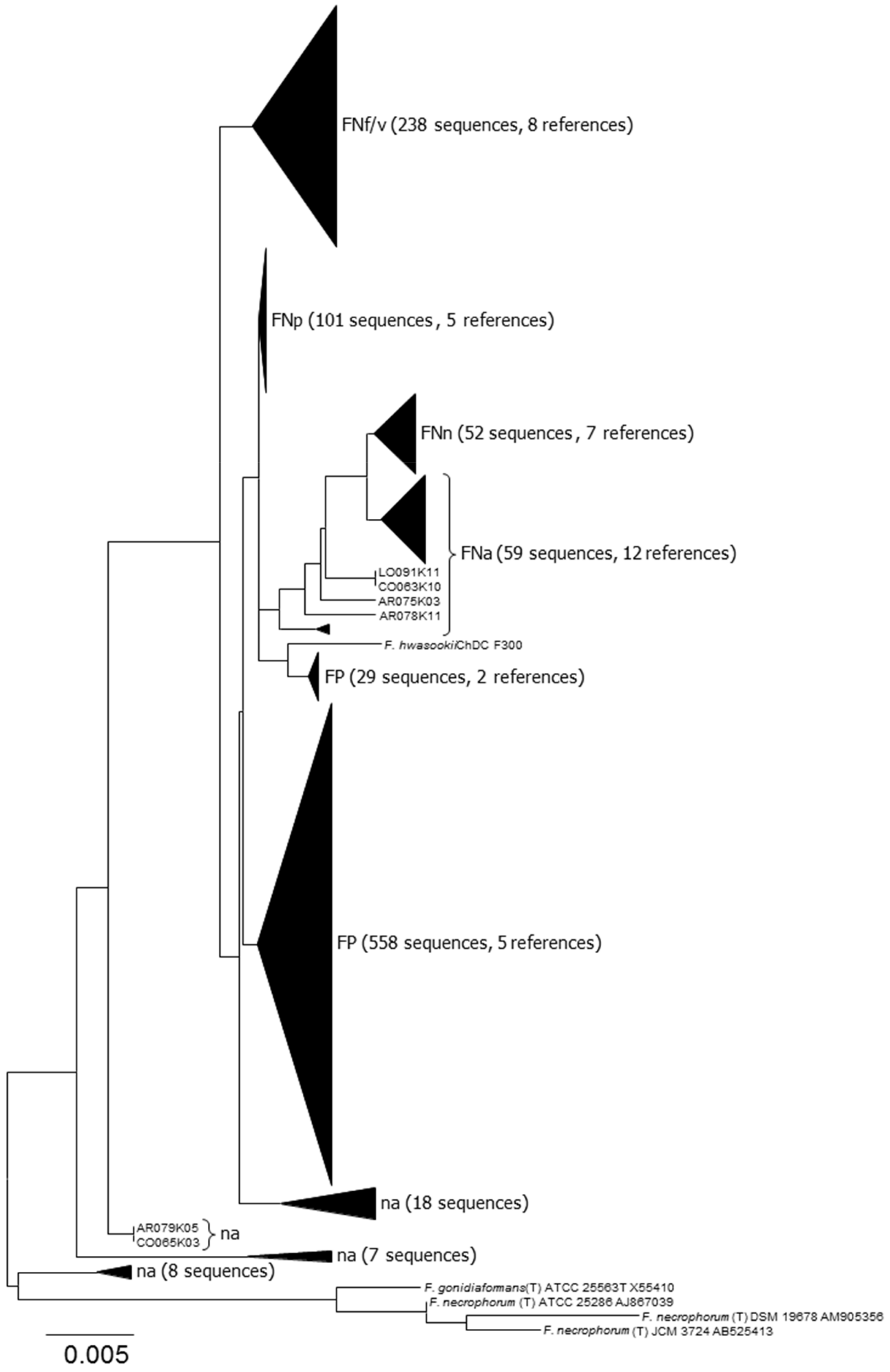

Supplementary Figure 2: Phylogenetic relationships among 16S rRNA gene types from this study and from those corresponding to known isolates of *F. nucleatum* (FN) subspecies and *F. periodonticum* (FP) along with other members of the genus *Fusobacterium*. The majority of own sequences (i.e. 1,037 out of a total of 1,072 sequences) could be assigned to distinct subspecies. The remaining 35 sequences indicated with "na" (= not assigned) have not been used for further downstream analysis. Where possible, sequences have been grouped in triangles for transparency. The numbers refer to the numbers of sequences in those triangles plus the number of reference sequences. Reference sequences are those shown and described in Figure 1 of the main article. FNf/v: *F. nucleatum* subsp. *fusiforme*/*vincentii*; FNp: *F. nucleatum* subsp. *polymorphum*; FNa: *F. nucleatum* subsp. *animalis*; FNn: *F. nucleatum* subsp. *nucleatum*. The scale bar corresponds to 0.005 substitutions per nucleotide.
